# Assessing Adherence to Royal College of Surgeons Guidelines: A Closed-Loop Audit of Operation Notes in a Tertiary Healthcare Unit

**DOI:** 10.7759/cureus.45743

**Published:** 2023-09-21

**Authors:** Muhammad Jawad Zahid, Aarzish Ijaz, Wagma Hidayat, Mahnoor Ataullah Jan, Hamza Rafi, Haq Nawaz, Haider Khan

**Affiliations:** 1 Department of Surgery, Hayatabad Medical Complex Peshawar, Peshawar, PAK; 2 Department of Physiology, Northwest School of Medicine, Peshawar, PAK; 3 Department of General Surgery, Hayatabad Medical Complex Peshawar, Peshawar, PAK

**Keywords:** record-keeping practices, audit, rcs guidelines, surgical documentation, operation notes

## Abstract

Introduction

Accurate, comprehensive, and legible operation notes are essential for maintaining patient records, supporting healthcare professionals, and facilitating research. The study focused on adherence to Royal College of Surgeons (RCS) guidelines established in 2008. Despite the guidelines, poor documentation practices have been reported globally. This audit seeks to address this issue and enhance documentation quality.

Methodology

The audit evaluated 19 parameters as defined in the 2014 RCS operative note guidelines. Data collection occurred during the initial cycle, spanning from March to April 2023, encompassing all surgical procedures at Hayatabad Medical Complex (HMC). Subsequently, a re-audit took place in July 2023 to gauge enhancements following a survey and educational intervention that took place in June 2023. The process included the formation of an audit team, securing ethical approval, and implementing a comprehensive methodology for data collection and analysis. The study spanned two data collection cycles to comprehensively assess improvements.

Results

Comparing initial and re-audit cycles (n = 390 and n = 108, respectively), improvements were observed in several documentation aspects. Parameters such as surgery date, elective/emergency classification, and names of key personnel showed significant enhancement. Notable improvements were also seen in the recording of operative details, complications, extra procedures, and post-operative care instructions. In our department, an educational survey was conducted to gain insights into compliance rates. This survey underscored the significance of adhering to RCS guidelines, identified the factors influencing adherence, and proposed strategies for improvement.

Conclusion

The audit affirmed the significance of adhering to RCS guidelines for operation note documentation. The study demonstrated improvements in documentation practices, emphasising the importance of accurate records for patient care, research, and ethical standards. The findings validate RCS guidelines as a tool for the identification of defects in documentation and thus as a guide that highlights where improvements are necessary. Addressing challenges identified in this audit can drive the department towards becoming a model for RCS guideline adherence and showcasing high-quality surgical documentation and patient-centred care.

## Introduction

The provision of high-quality healthcare depends on the accurate and timely maintenance of clinical records [1]. The Royal College of Surgeons (RCS) (England) also places significant emphasis on the necessity for surgeons to uphold accurate, comprehensive, and legible records of patient interactions, contemporaneously [2]. The efficient maintenance of medical records assumes a pivotal role in patient care, fostering a systematic assessment of patient data that aids in the creation of effective treatment strategies. In modern healthcare, adept record-keeping is imperative, especially when addressing concerns related to medical malpractice [3-5]. These notes not only act as crucial reference tools for healthcare practitioners dealing with patients' surgical histories but also hold potential relevance in legal proceedings [3,6,7].

Moreover, these operative theatre (OT) notes possess dual significance within medical records, as they serve as invaluable resources for researchers. Globally, numerous studies have been conducted to assess the accuracy, completeness, validity, legibility, reliability, and precision of medical and OT notes. These investigations consistently expose subpar record-keeping practices [7-10].

In response to this challenge, the RCS (England) introduced operational note documentation guidelines in 2014 [2,11]. However, the adherence to these guidelines within our institution remains uncertain. Our primary goal is to evaluate the adherence to RCS guidelines for operation note documentation within our institution. We aim to identify areas where compliance may be improved and provide recommendations for refining note-keeping practices. While our study does not explicitly seek to validate the RCS guidelines in terms of improved outcomes, it underscores the importance of adherence to established standards in maintaining accurate and reliable documentation. This, in turn, contributes to enhancing the quality of patient care and lays the groundwork for future advancements in research within this field.

## Materials and methods

Ethical approval and study setting

The audit was carried out at the Department of Surgery, Hayatabad Medical Complex (HMC), Peshawar, Pakistan. Ethical approval was obtained from the HMC Ethical Review Board in May 2023, ensuring compliance with ethical guidelines and standards.

Study parameters and guidelines

The study aimed to assess the surgical practices at HMC based on 19 parameters outlined in the 2014 operative note guidelines for good surgical practice established by the RCS [8]. These parameters provided a comprehensive framework for evaluating various aspects of surgical documentation and care.

Data collection timeline

The retrospective data collection extended across two distinct phases: the initial phase from March to April 2023 and the post-implementation phase in July 2023. This comprehensive approach encompassed the entirety of surgical procedures undertaken at HMC during these specified periods. The assessment was all-encompassing, including both elective and emergency surgeries, thereby providing a comprehensive evaluation of surgical practices.

Data collection process

Data collection involved using a structured pro forma to document relevant information from operative notes. The pro forma covered essential details, such as procedure date and time, surgery type, operating surgeon's name, assistant, and theatre anaesthetist, operative procedure specifics, incision details, operative diagnosis, findings, complications, additional procedures with justifications, tissue manipulation specifics, closure technique, expected blood loss, antibiotic usage, deep vein thrombosis (DVT) prophylaxis (if applicable), post-operative care instructions, and legibility of written operative notes. Each note was evaluated based on criteria such as handwriting clarity, use of legible terminology, and overall readability. This assessment allowed for the classification of notes into categories of poor or satisfactory legibility. Two independent assessors were involved in this process to ensure objectivity and minimise bias.

Audit team composition

An audit team was formed, comprising qualified medical professionals, auditors, and administrative staff. This diverse team brought together expertise from various disciplines, ensuring a well-rounded evaluation of surgical practices.

Audit process

a. Initial Data Collection

Data on surgical practices were collected from March to April 2023, providing a baseline assessment of current procedures.

b. Presentation and Deliberation

Findings from the initial data collection were presented to a local committee in May 2023. Necessary changes and improvements were discussed and deliberated upon, reflecting a collaborative approach to enhancing surgical practices.

c. Implementation of Changes

The proposed changes were implemented in June 2023, reflecting a proactive effort to address identified deficiencies. See the Discussion section to see what changes have been implemented.

d. Second Data Collection

A second round of data collection took place in July 2023 to evaluate the impact of the implemented changes on surgical practices.

e. Final Analysis and Presentation

The results of the second data collection were subjected to a comprehensive analysis. The findings were presented in another local meeting, providing a platform for sharing insights and fostering continuous improvement.

Data analysis

The chosen operative notes were meticulously assessed against the predefined audit criteria. Any deviations or deficiencies were systematically documented. Data analysis was conducted using Statistical Package for the Social Sciences (IBM SPSS Statistics for Windows, IBM Corp., Version 28.0, Armonk, NY). The chi-square test and Fisher's exact test, where appropriate, were used to calculate the p-values and determine significance.

## Results

Table 1 presents a comparison of baseline demographics between the initial audit cycle (n = 390) and the re-audit cycle (n = 108). In terms of age, the mean age in the initial audit cycle was 39.47 years (SD = 16.66), while the re-audit cycle showed a slightly higher mean age of 40.12 years (SD = 14.94). The gender distribution also differed, with the initial cycle having 51.3% male and 48.7% female participants, whereas the re-audit cycle had 47.3% male and 52.7% female participants. Analysing the American Society of Anesthesiologists (ASA) Grade distribution, the initial cycle displayed 35.3% Grade I, 19.7% Grade II, 8.2% Grade III, 1.04% Grade IV, and 35.6% not recorded, while the re-audit cycle showed 51.8% Grade I, 17.6% Grade II, 13.9% Grade III, 0.9% Grade IV, and 15.9% not recorded. While the re-audit cycle had a shorter duration (one month compared to two months) and thus fewer participants, it still revealed differences in age, gender distribution, and ASA Grade when compared to the initial audit cycle.

**Table 1:** Baseline characteristics of the initial audit cycle and the re-audit cycle ASA: American Society of Anesthesiologists, PGY: postgraduate year

Variable	Initial Audit Cycle (n = 390)	Re-audit Cycle (n = 108)
Age (Mean SD)	39.47 (16.66)	40.12 (14.94)
Gender		
Male	200 (51.3)	51 (47.3)
Female	190 (48.7)	57 (52.7)
ASA Grade		
I	138 (35.3)	56 (51.8)
II	77 (19.7)	19 (17.6)
III	32 (8.2)	15 (13.9)
IV	4 (1.04)	1 (0.9)
V	0	0
Not Recorded	139 (35.6)	17 (15.9)
Recorded By		
PGY I	100 (25.6)	14 (12.9)
PGY II	80 (20.5)	20 (18.4)
PGY III	22 (5.6)	26 (24)
PGY IV	148 (37.9)	37 (34.2)
Consultant	40 (10.3)	11 (10.1)

Initial audit cycle findings

In the initial audit, 390 operative notes were evaluated based on the RCS guidelines, revealing areas for improvement in documentation. While the date of surgery was well-recorded in 97.4% of cases, information about the time of surgery was significantly lacking, with only 3.6% of surgeries having this detail documented. Similarly, the classification of surgeries as elective or emergency was poorly documented, with only 3.6% of cases appropriately classified.

The names of the operating surgeon and assistant were well-documented for 99% of surgeries, but there was room for improvement in recording the name of the theatre anaesthetist, which was documented in 86.7% of cases. Most operative details, such as procedures (95.9%), findings (96.9%), and the surgeon's signature (88.2%), were well-documented. However, certain aspects, like the operative diagnosis (6.2%) and identification of any used prostheses (5.1%), were documented in a relatively small proportion of cases.

Re-audit cycle findings

In the re-audit cycle, improvements in documentation were evident. The date of surgery achieved 100% compliance, indicating an enhancement in this aspect. Similarly, the time of surgery was recorded in an impressive 91.6% of cases. The classification of surgeries as elective or emergency showed remarkable improvement, reaching 100% compliance.

Documentation of important personnel, including the operating surgeon, assistant, and theatre anaesthetist, demonstrated 100% compliance. Additionally, operative procedure details (100%) and operative findings (100%) were comprehensively recorded. Parameters that experienced a considerable boost in the documentation included any problems or complications during surgery (98.1%) and any extra procedures performed for reasons (88.89%). Furthermore, detailed post-operative care instructions achieved 100% compliance.

Differences between the initial audit and re-audit cycle

A comparison between the two audit cycles reveals significant improvements in documentation practices. The re-audit cycle showcased remarkable progress, with several parameters achieving full compliance, including the time of surgery, elective/emergency classification, and the names of key personnel. Additionally, the comprehensive recording of operative details and post-operative care instructions improved significantly. The overall re-audit cycle demonstrated the efficacy of the RCS guidelines in enhancing documentation practices, leading to a more standardised and comprehensive recording of essential surgical parameters (Table 2).

**Table 2:** Frequency of documentation of the parameters identified in the Royal College of Surgeons guidelines

Parameter (n,%)	Initial Audit (n = 390)	Re-Audit (n = 108)	Difference (%)	P-value
Date of Surgery	380 (97.4)	108 (100)	2.6	0.004
Time of Surgery	14 (3.6)	99 (91.6)	88	0.023
Elective/Emergency	14 (3.6)	108 (100)	96.4	0.912
Names of the operating surgeon and assistant	386 (99)	108 (100)	1	0.410
Name of theatre anesthesiologist	338 (86.7)	108 (100)	13.3	0.093
Operative procedure	374 (95.9)	108 (100)	4.1	<0.001
Incision Name	348 (89.2)	108 (100)	10.8	0.102
Operative Diagnosis	24 (6.2)	92 (85.1)	78.9	<0.001
Operative findings	278 (96.9)	108 (100)	3.1	0.040
Any problems or complications	26 (6.7)	106 (98.1)	91.4	0.403
Any extra procedure performed and the reason why it was performed	44 (11.3)	96 (88.89)	77.59	0.314
Details of tissue removed added or altered	32 (8.2)	86 (79.6)	71.4	0.023
Identification of any prosthesis used, including serial numbers of the prosthesis and other implant materials	20 (5.1)	40 (37)	31.9	0.002
Details of closure technique	60 (15.4)	107 (99.07)	83.67	<0.001
Anticipated blood loss	8 (2.1)	100 (92.5)	90.4	0.701
Antibiotic prophylaxis (if applicable)	0	104 (96.3)	96.3	1.14
Venous Thromboembolism prophylaxis (If applicable)	0	106 (98.1)	98.1	0.820
Detailed post-operative care instructions	184 (47.2)	108 (100)	52.8	<0.001
Signature	344 (88.2)	108 (100)	11.8	<0.001
Legibility of Notes				
Satisfactory	296 (75.9)	107 (99.07)	23.17	0.003
Poor	94 (24.1)	1 (0.9)	-23.2	

After the initial audit cycle, an educational survey was conducted to gain insight into the surgeon's perspective regarding the lack of compliance with the RCS guidelines on recording operative notes. The aim of this survey was to better understand the reasons behind this non-compliance and to come up with effective strategies to improve the current practices. The findings of the survey are given in Table 3.

**Table 3:** Educational survey findings from 63 respondents PGY: postgraduate year

Variable (n,%)	Total (n=63)
Respondent’s Level of Education/Training	
Consultant	13 (20.6)
Registrar	2 (3.2)
Resident PGY IV	7 (11.1)
Resident PGY III	11 (17.5)
Resident PGY II	15 (23.8)
Resident PGY I	15 (23.8)
Operative notes should be recorded according to the RCS guidelines	61 (96.8)
How do operation notes affect patient care?	
Follow-up of patients	45 (71.4)
Prevention of future mismanagement	41 (65.1)
Planning of future interventions	36 (57.1)
Communication with nurses and physicians	31 (49.2)
Reason for non-compliance	
Absence of formal teaching	37 (58.7)
Incomplete operation note format	13 (20.6)
Lack of time	13 (20.6)
In what way did you learn how to write an operation note?	
learned by reading other operation notes	38 (60.3)
learned by practice	19 (30.2)
Formally Trained on operation note-writing	6 (9.5)
Measures for improvement of quality and completeness of the operation notes	
Formal education	43 (63.8)
Update of operation note proforma	39 (61.9)
Aide Mémoire in Opera Theatre	19 (30.2)

The table presents findings from an educational survey involving 63 respondents and focuses on various aspects of operation note-writing in the medical context. The respondent's level of education/training is detailed, with the majority being residents across different years (PGY I to IV), consultants, and registrars. The survey highlights that a significant percentage of respondents (96.8%) emphasise adherence to RCS guidelines for recording operative notes. The impact of operation notes on patient care is explored, with follow-up of patients (71.4%), prevention of future mismanagement (65.1%), and planning future interventions (57.1%) being identified as key factors. Non-compliance reasons are indicated, including absence of formal teaching, incomplete note format, and lack of time. Respondents primarily learned operation note-writing through reading other notes (60.3%) and practice (30.2%), with a smaller portion having formal training (9.5%). Strategies for improving note quality and completeness involve formal education, updating the note pro forma, and using an Aide Mémoire in the OT.

## Discussion

**Figure 1:**
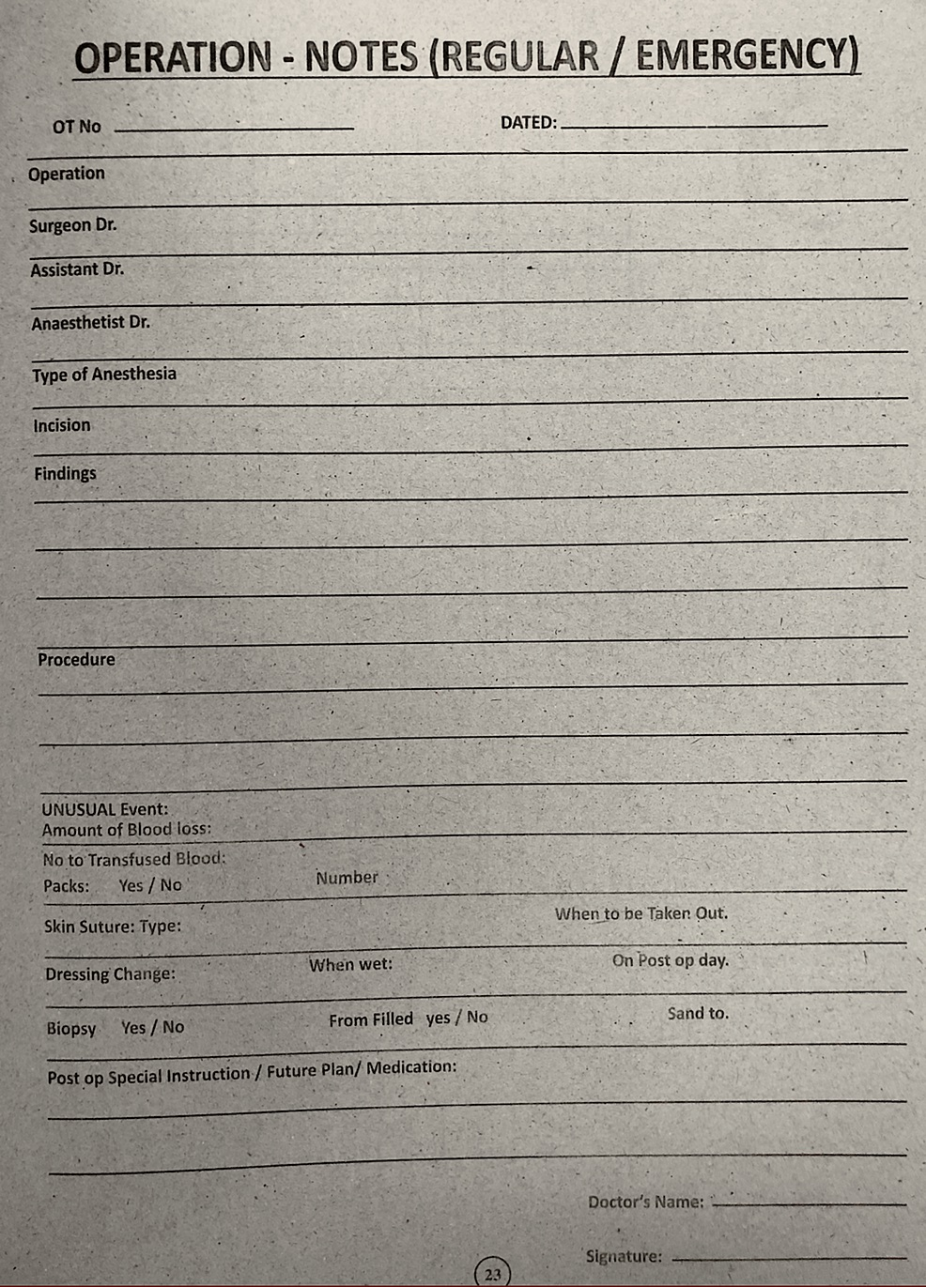
Operational notes pro forma, which were utilized prior to putting the first audit's recommendations into practice.

The initial audit of operative notes revealed deficiencies in the documentation process, notably in the operative notes pro forma (Figure 1), and a lack of clear standards for proper documentation. Subsequent to the audit, these findings were presented to the Department of Surgery, leading to several constructive recommendations.

**Figure 2:**
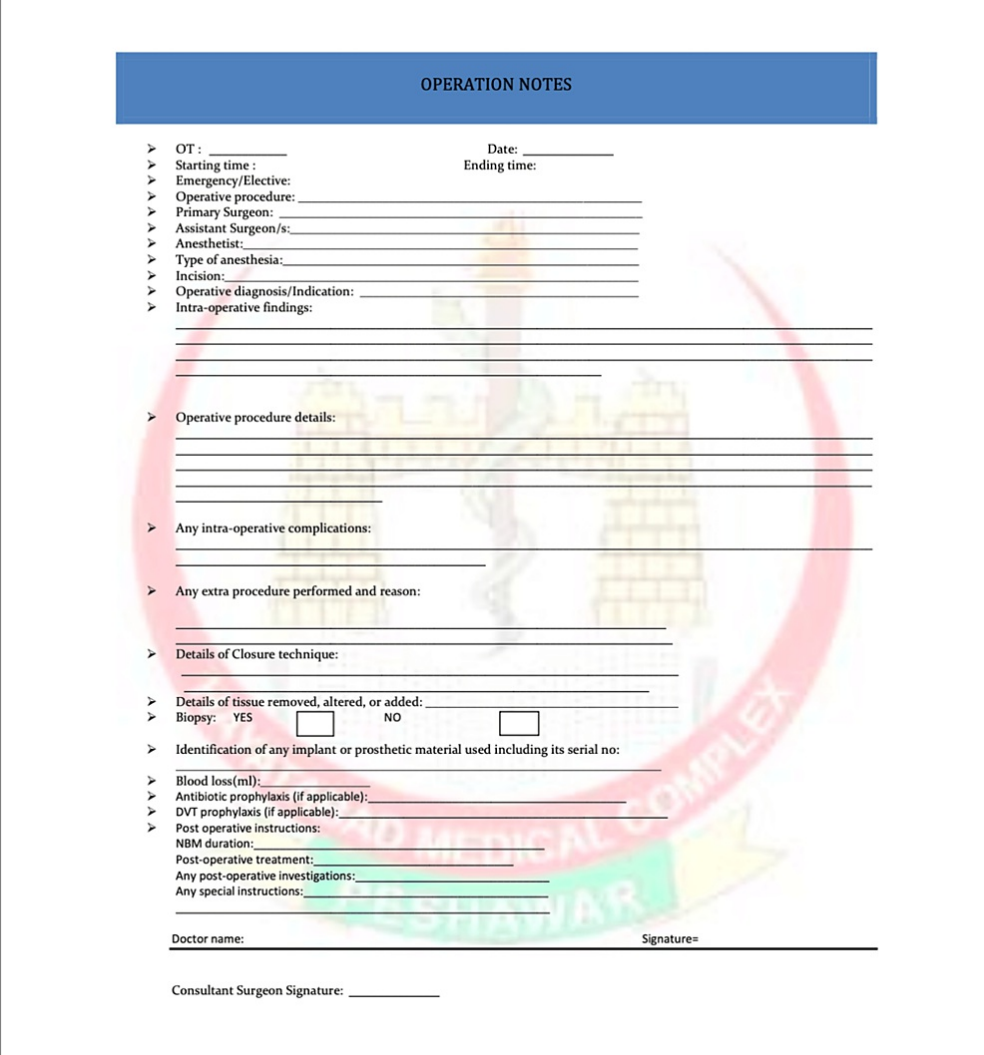
Updated operative notes pro forma that were revised and utilized for documentation following the initial audit.

The redesign of the operative notes pro forma (Figure 2) aimed to address these shortcomings by introducing additional parameters missing in the initial notes. These encompassed surgery time, operative diagnosis, and any supplementary procedures undertaken with reasons, complications, alterations in tissue details, identification of utilised prosthetics along with their serial numbers, specifics of closure technique, and provisions for antibiotic prophylaxis (if applicable) and venous thromboembolism prophylaxis (if applicable).

In light of the survey, several other noteworthy suggestions were put forth. These encompassed the introduction of formal education for proficient operative note composition, advocating senior residents as primary writers with endorsement by the consultant surgeon. To promote optimal practices, an Aide Mémoire detailing operative note guidelines was prominently displayed in the OT. Surgeons were encouraged to promptly record surgical notes post-procedure to enhance precision. Another key aspect involved making it obligatory for consultant surgeons to review and approve operative notes completed by residents. Implementation of these recommendations yielded positive outcomes. Ultimately, the establishment of a biannual audit cycle was proposed to ensure the enduring effectiveness of these enhancements in practice.

During the initial audit cycle, the parameters lacking in the surgical notes pro forma exhibited the lowest adherence. However, after the introduction of the revised pro forma, the second phase of the audit demonstrated significant improvement. Parameters such as operative diagnosis, complications, details of additional procedures, specifics of tissue manipulation, prosthesis identification, closure technique details, anticipated blood loss, and antibiotic and thromboembolism prophylaxis achieved compliance rates of 85%, 98%, 88%, 79%, 37%, 99%, 92%, 96%, and 98%, respectively. Our study findings align with similar investigations [3,12-14] where the implementation of a revised surgical pro forma led to a substantial increase in adherence to RCS guidelines.

The subsequent phase of the audit cycle yielded highly positive results. When compared to the initial phase, compliance with certain parameters, such as the surgeon's and assistant's names, and details of the operative procedure, reached a full 100%. These findings are consistent with similar studies [7,15-17], where compliance rates were also reported at 99%. Additionally, the recording of the surgery date, time, and classification as elective or emergency achieved compliance rates of 100%, 91%, and 100%, respectively [7].

During the re-audit cycle, parameters such as the name of the anaesthetist, incision details, operative procedure specifics, and detailed postoperative instructions demonstrated full adherence to RCS guidelines, each achieving a compliance rate of 100%. Our findings are in line with a similar study by Singh R et al. [18] and can be attributed to the formal training provided to general surgical residents as well as the introduction of the Aide Mémoire in the OTs.

In the modern healthcare landscape, the concept of clinical governance has emerged as a pivotal element, playing an indispensable role in the provision of top-notch medical services. The imperative nature of quality enhancement endeavours cannot be understated, as they lay the foundation for the delivery of exceptional patient care. Through a meticulously executed closed-loop audit, we unveiled a noteworthy upturn in standards and methodologies, achieved through the iterative process of re-auditing. This resonates harmoniously with the observations of fellow researchers, who have also documented parallel advancements in the realm of operative note documentation upon conducting subsequent audits [7,10,15]. These congruent findings stand as a testament to the effectiveness of continuous assessment and improvement practices in elevating the quality of healthcare delivery.

## Conclusions

This closed-loop audit had a primary focus on evaluating adherence to RCS guidelines for operation note documentation. It not only provided valuable insights into note-keeping practices but also offered recommendations to elevate the quality of documentation. The study underlines the pivotal role of accurate notes in patient care, research, and upholding ethical standards. The documented improvements not only validate the effectiveness of RCS guidelines in guiding note-taking but also pinpoint areas where interventions are necessary to enhance documentation quality. The audit's success is exemplified by its potential to drive positive transformation, resulting in improved patient outcomes and the advancement of surgical practices through standardised documentation.
